# Full-Face Mask Use during SCUBA Diving Counters Related Oxidative Stress and Endothelial Dysfunction

**DOI:** 10.3390/ijerph19020965

**Published:** 2022-01-15

**Authors:** Morgan Levenez, Kate Lambrechts, Simona Mrakic-Sposta, Alessandra Vezzoli, Peter Germonpré, Hadrien Pique, Fabio Virgili, Gerardo Bosco, Pierre Lafère, Costantino Balestra

**Affiliations:** 1 Environmental, Occupational, Aging (Integrative) Physiology Laboratory, Haute Ecole Bruxelles-Brabant (HE2B), 1180 Brussels, Belgium; mlevenez@he2b.be (M.L.); klambrechts@he2b.be (K.L.); pgermonpre@gmail.com (P.G.); phadrien98@gmail.com (H.P.); plafere@he2b.be (P.L.); 2 Institute of Clinical Physiology, National Research Council (IFC-CNR), Piazza dell’Ospedale Maggiore, 20162 Milano, Italy; simona.mrakicsposta@cnr.it (S.M.-S.); alessandra.vezzoli@cnr.it (A.V.); 3 Hyperbaric Centre, Queen Astrid Military Hospital, 1120 Brussels, Belgium; 4 DAN Europe Research Division, Contrada Padune, 64026 Roseto, Italy; 5 Council for Agricultural Research and Economics—Food and Nutrition Research Centre (CREA-AN), Via Ardeatina 548, 00187 Rome, Italy; 6 Environmental Physiology & Medicine Laboratory, Department of Biomedical Sciences, University of Padova, 35122 Padova, Italy; gerardo.bosco@unipd.it; 7 Physical Activity Teaching Unit, Motor Sciences Department, Université Libre de Bruxelles (ULB), 1050 Brussels, Belgium

**Keywords:** hypoxia, hyperoxia, hyperbaric breathing, nitric oxide, vascular reactions, breathing, extreme environments

## Abstract

Impaired flow mediated dilation (FMD), an index of vascular stress, is known after SCUBA diving. This is related to a dysfunction of nitric oxide (NO) availability and a disturbance of the redox status, possibly induced by hyperoxic/hyperbaric gas breathing. SCUBA diving is usually performed with a mask only covering “half face” (HF) and therefore forcing oral breathing. Nasal NO production is involved in vascular homeostasis and, as consequence, can significantly reduce NO possibly promoting vascular dysfunction. More recently, the utilization of “full-face” (FF) mask, allowing nasal breathing, became more frequent, but no reports are available describing their effects on vascular functions in comparison with HF masks. In this study we assessed and compared the effects of a standard shallow dive (20 min at 10 m) wearing either FF or a HF mask on different markers of vascular function (FMD), oxidative stress (ROS, 8-iso-PGF2α) and NO availability and metabolism (NO_2_, NOx and 3-NT and iNOS expression). Data from a dive breathing a hypoxic (16% O_2_ at depth) gas mixture with HF mask are shown allowing hyperoxic/hypoxic exposure. Our data suggest that nasal breathing might significantly reduce the occurrence of vascular dysfunction possibly due to better maintenance of NO production and bioavailability, resulting in a better ability to counter reactive oxygen and nitrogen species. Besides the obvious outcomes in terms of SCUBA diving safety, our data permit a better understanding of the effects of oxygen concentrations, either in normal conditions or as a strategy to induce selected responses in health and disease.

## 1. Introduction

Recreational SCUBA (self-contained underwater breathing apparatus) diving is considered a safe leisure activity. However, it is known to induce (generally mild) adaptive responses potentially interfering with different physiological pathways, some of them being possibly harmful. When SCUBA divers stay at depth, they are exposed to inert gas supersaturation that may be translated into vascular gas emboli (VGE) during and following ascent while decompressing [1]. Although decompression-induced bubble formation is a pivotal event in decompression sickness (DCS), the exact pathophysiological mechanisms linking VGE to DCS are still unclear; however, both are linked and evidently related to ambient pressure drop.

Changes in flow mediated dilation (FMD), a sensitive marker of a physiological response of the vascular system, has been repeatedly reported to occur after diving, suggesting vascular dysfunction of large conductance arteries [2,3,4]. This dysfunction, which is usually recovered to normality a few hours after surfacing, is however to be considered an index of vascular stress. More recently, diving has been reported to induce a response extended to the microvascular endothelium [2] and to the macro and microvascular smooth muscle [2,5]. The predive administration of bioactive molecules belonging to a very large family known as polyphenols and frequently reported as having a putative “antioxidant capacity” have been reported to partially prevent postdive alterations of FMD [6,7,8], suggesting the implication of an oxidative stress in this response. Indeed, impaired FMD [4,9] and increased oxidative stress [7,10] were also reported postdive even in the absence of VGE, suggesting that the organism casts out a physiological response to high oxygen, which does not necessarily result in a clinically relevant dysfunction. It seems also plausible that other factors contribute to postdive alteration of vascular function [11]. For instance, it has been shown that circulating bubbles can lead to vascular dysfunction [12] either by a direct “mechanical” contact between circulating bubbles and endothelial cells [13,14] or by an uncontrolled activation of the coagulation cascade [15,16,17], or by a combination of these two mechanisms. Activation of haemostatic pathways contributes to the postdive increase of circulating microparticles, which in turn leads to leukocytes activation [18,19] and their adhesion to the endothelium [20].

For several years, preconditioning strategies to reduce this “decompression stress” have been considered and investigated [21]. Nutritional strategies with polyphenols-rich food supplementation (dark chocolate and red orange complex or vitamin C, to name a few) have been shown to reduce vascular dysfunction [8,22]. Physical interventions (predive vibration, oxygen breathing, exercise, sauna) appear capable in limiting the generation of VGE after a dive through the reduction of pre-existing gas micronuclei [21]. Two decades ago, specific attention and focus was given to nasal breathing and vascular dysfunction. In fact, nitric oxide production by the nasal fossae and sinuses mucosae is an important variable involved in vascular homeostasis and, as a consequence, oral breathing can significantly reduce circulating nitric oxide (NO), possibly promoting vascular dysfunction [23]. Some specific abnormalities can interfere with normal NO production by the nasal mucosae and a lead to discomfort or even some surgical interventions; none of our subjects declared such abnormalities [24,25] during the eligibility discussion.

In this study we aimed to investigate the role of nasal breathing on vascular function occurring after diving, starting from the observation that all previously available studies in diving have been conducted using oral breathing, with conventional “half-face” masks, and none utilizing nasal breathing by means of the so-called “full-face” masks. To limit confounding factors, we specifically planned an experimental design based on a known “bubble free” dive profile.

## 2. Materials and Methods

### 2.1. Experimental Protocol

**Figure 1:**
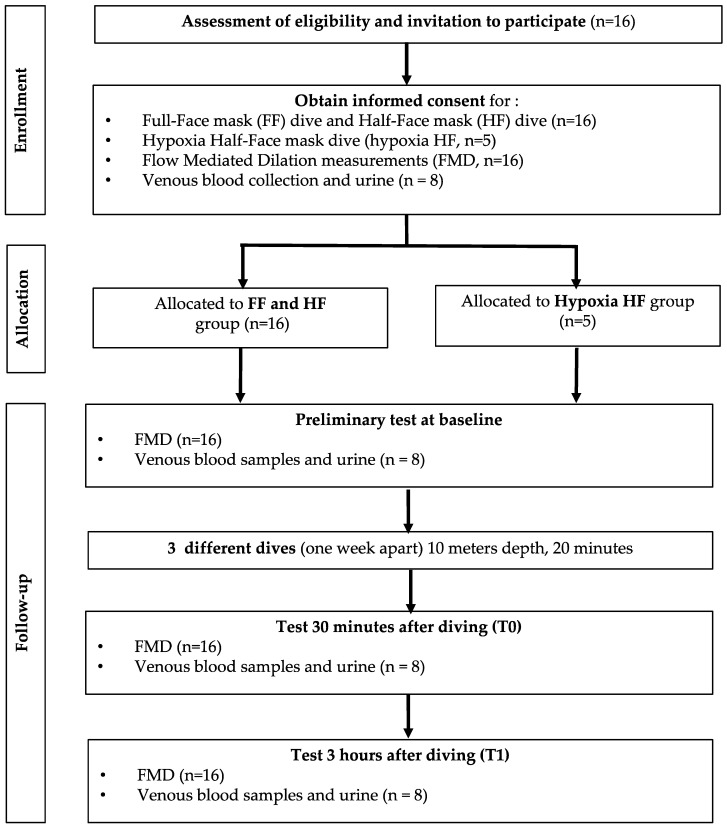
Flowchart of the experimental protocol.

After written informed consent, 16 (6 females and 10 males) (Figure 1) healthy nonsmoking divers (minimum certification ‘‘Autonomous Divers’’ according to European norm EN 14153-2 or ISO 24801-2 with at least 50 logged dives) volunteered for this study. None of them had a history of previous cardiac abnormalities or were under any cardio- or vaso-active medication. They were selected from a large population of divers in order to have a homogenous sample: aged 44.7 ± 12.4 years old (mean ± SD); height 173 ± 6.6 cm; weight 75.2 ± 13.7 kg.

All experimental procedures were conducted in accordance with the Declaration of Helsinki [26] and approved by the Ethics Committee approval from the Bio-Ethical Committee for Research and Higher Education, Brussels (N° B200-2020-088).

**Figure 2:**
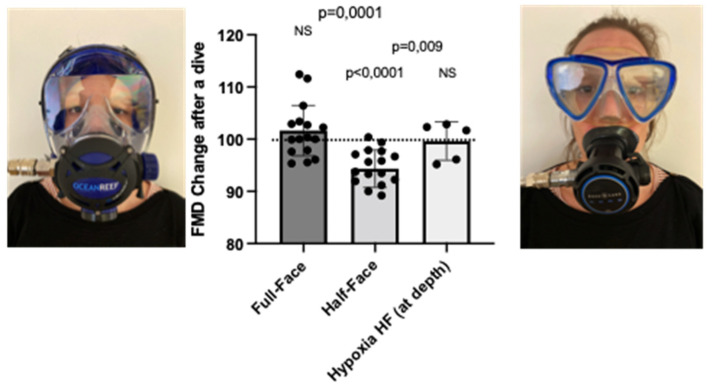
Flow mediated dilation (FMD) changes after a dive (10 m for 20 min) either wearing a full-face mask or a classical “half-face” diving mask (*n* = 16). In hypoxic dives (*n* = 5), subjects were breathing a gas mixture providing 0.16 ATA of PO_2_ at depth. FMD changes are expressed as % of predive values, every diver acting as his own control. Results are expressed as percentage (mean ± SD); NS = nonsignificant.

Participants were prospectively randomized into two groups of 8 persons each. They were assigned to either “full-face mask” (Ocean Reef, Neptune Space G-Divers IDM mask, see Figure 2) or “half-face” (usual diving mask, see Figure 2) mask group for the first dive, and vice versa for the second one, making each diver his own control. Another specific experiment was also performed (*n* = 5) using a usual diving mask but breathing a gas mixture corresponding to 16% of oxygen at depth, to reduce oxygen reactive species inhalation. This mixture was a hypoxic trimix composed of 8% oxygen mixed with helium and nitrogen (Trimix); this mixture, being hypoxic at surface, can only be breathed at depth, the divers were using air during their descent and their ascent, these two portions of the dive took around one minute each, the slightly hypoxic breathing took 20 min.

All dives were performed in a pool environment dedicated to SCUBA diving (NEMO 33, Brussels, Belgium). The dive profile was specifically chosen to avoid any bubble formation (10 m depth, 20 min). Indeed, this dive does not require any decompression procedure as for every decompression table actually accepted the no-decompression time for a depth of 10 m is far over 200 min. To be sure, we chose 10 times less. Additionally, the postdive bubble measurements as previously reported were “no-bubble” using the same bubble measurements protocol performed by our group, see Balestra et al. 2016 [27].

However, divers were submitted to a significant oxidative stress in order to interfere with the endothelial function since at that depth, oxygen inspired fraction is close to 40% (0.4 PO_2_ ± 300 mmHg).

### 2.2. Flow Mediated Dilation (FMD)

FMD, an established measure of the endothelium-dependent vasodilation mediated by nitric oxide (NO) [28], was used to assess the effect of diving on main conduit arteries. Subjects were at rest for 15 min in a supine position before the measurements were taken; they were asked not to drink caffeinated beverages for 6 h preceding measurements. Subjects were instructed not to perform strenuous physical exercise 24 h before or stay in altitude up to 2 weeks before and during the entire study protocol. Brachial artery diameter was measured by means of a 5.0–10.0 MHz linear transducer using a Mindray DP-30 digital diagnostic ultrasound system immediately before and 1 min after a 5 min ischemia induced by inflating a cuff placed on the forearm to 180 mmHg, as described previously [29].

All ultrasound assessments were performed by an experienced operator with more than 100 scans/year, which is recommended to maintain competency with the FMD method [30].

When the images were chosen for analysis, the boundaries for diameter measurement were identified manually with an electronic caliper (provided by the ultrasonography software) in a threefold repetition pattern to calculate the mean value. In our laboratory, the mean intra observer variability for FMD measurement for the operator (KL) recorded the same day, on the same site and on the same subject, was 1.2 ± 0.2%.

Post-dive values were obtained 20–30 min after surfacing. FMD was calculated as the percent increase in arterial diameter from the resting state to maximal dilation.

### 2.3. Venous Blood Samples and Urine

Among the 16 participants, 8 accepted venous blood collection. Venous blood samples were collected at baseline (before diving), 30 min (T0) and 3 h (T1) after diving. Fifteen milliliters of blood were collected in EDTA and heparinized vacutainer tubes for separation of plasma. Urine was collected by voluntary voiding in a sterile container and stored in multiple aliquots at −20 °C. All samples were stored in multiple aliquots at −80 °C until assayed and performed within one month from the collection.

### 2.4. Blood Sample Analysis

#### 2.4.1. ROS Production

EPR, X-band instrument (E-Scan—Bruker BioSpin, GmbH, Ettlingen, Germany) was adopted. ROS production rate was determined by means of a well-consolidated method in blood [31,32,33,34]. Spin probe, CMH (1-hydroxy-3-methox-ycarbonyl-2,2,5,5-tetramethylpyrrolidine) was used for determination, and stable radical CP· (3-Carboxy2,2,5,5-tetramethyl-1-pyrrolidinyloxy) was used as external reference to convert ROS determinations in absolute quantitative values (μmol·min^−1^). Samples were stabilized at 37 °C by ‘‘Bio III’’ controller unit, interfaced to the spectrometer.

#### 2.4.2. 8-Isoprostane

Lipid peroxidation was assessed in urine by competitive immunoassay (Cayman Chemical, Ann Arbor, MI, USA) measuring 8-isoprostane (8-iso-PGF2α) concentration following the manufacturer’s recommendations. The 8-iso-PGF2α concentrations were determined using a standard curve. Samples and standards were spectrophotometrically read at a wavelength of 412 nm.

#### 2.4.3. Nitric Oxide Metabolites (NOx)

NOx concentrations were determined for urine via a colorimetric method based on the Griess reaction, using a commercial kit (Cayman Chemical, Ann Arbor, MI, USA) as previously described [35,36]. Samples were spectrophotometrically read at 545 nm.

#### 2.4.4. Inducible Nitric Oxide Synthase (iNOS)

To assess inducible nitric oxide synthase (iNOS) expression in plasma, a human NO2/iNOS ELISA kit (catalog number EH0556; FineTest, Wuhan, China) was used. This assay was based on sandwich enzyme-linked immune-sorbent assay technology. NOS2/iNOS protein synthesis was determined using a standard curve.

#### 2.4.5. Nitrotyrosine (3-NT)

The concentration of 3-NT on plasma was measured by competitive-ELISA method, using an assay kit (catalog number EU2560; FineTest, Wuhan, China). The analysis was carried out in accordance with the manufacturer’s instructions, and the 3-NT concentration was measured spectrophotometrically at a wavelength of 450 nm by comparing the sample OD (optical density) to a standard curve.

All the samples and standards were read by a microplate reader spectrophotometer (Infinite M200, Tecan Group Ltd., Maännedorf, Switzerland). The determinations were assessed in duplicate, and the interassay coefficient of variation was in the range indicated by the manufacturer.

### 2.5. Statistical Analysis

Normality of data was performed by means of Shapiro–Wilk or D’Agostino–Pearson tests. When a Gaussian distribution was assumed, data were analyzed with a one-way ANOVA for repeated measures with Dunnett’s post hoc test; when comparisons were limited to two samples, a paired or nonpaired *t*-test was applied. If the Gaussian distribution was not assumed, the analysis was performed by means of a nonparametric multiple comparisons Dunn’s test. Taking the baseline measures as 100% (percentage or fold change), changes were calculated for each diving protocol, allowing an appreciation of the magnitude of change rather than the absolute values. All statistical tests were performed using a standard computer statistical package, GraphPad Prism version 5.00 for Windows (GraphPad Software, San Diego, CA, USA). A threshold of *p* < 0.05 was considered statistically significant. All data are presented as mean ± standard deviation (SD). Sample size was calculated by setting the power of the study at 95% and assuming that variables associated with diving would have been affected on a similar extent than that observed in our previous studies [10].

## 3. Results

### 3.1. Nasal–Oral Breathing by Wearing a Full-Face Mask and Breathing a Hypoxic Air Mixture Prevents FMD Reduction after a Shallow Dive, in Comparison to a Half-Face Mask

We previously demonstrated that diving is associated with a significant reduction of NO-related endothelial function of large arteries, and that predive administration of polyphenol-rich food items (chocolate) or plant extract from red orange significantly counters this response [8,22]. The utilization of full-face masks during SCUBA diving allows breathing through the nose, which is a confirmed physiological strategy to increase circulating NO [8], therefore potentially modulating the effects of diving on FMD. Accordingly, FMD variation values were 102 ± 6.7% (*p* = 0.168, NS = nonsignificant) and 94.3 ± 3.6% (*p* < 0.0001) when utilizing a full-face mask and a half-face mask (regular mask), the difference between both conditions being highly significant (*p* = 0.001) (Figure 2). FMD returned to normal values 3 h after half mask exposure and remained stable after full-face exposure; these values were, respectively, 100.6 ± 2.5% and 100.2 ± 2.8%.

The hypoxic dive experiment was performed on 5 subjects (4 male and 1 female) out of the divers that already performed the set of dives described above, using a regular (half-face) mask (and therefore breathing only orally). This experiment would let the diver breath at depth a hypoxic mixture (FiO_2_ of 8% corresponding to a PO_2_ of 0.16 ATA) corresponding to 16% of oxygen at surface, thus reducing the absolute inspired pressure of oxygen, significantly reducing, in theory, the oxidative stress associated with high oxygen.

In this case, we observed no difference in postdive FMD variations in subjects with a half-face mask (breathing hypoxic mixture at depth) directly postdive 99.6 ± 3.6 %, after 30 min (98.8 ± 0.7% relative to predive values), neither after 3 h (99.6 ± 0.6%). Comparing this with the significant difference observed in the same subjects when in hyperoxic conditions (half-face mask, breathing air at depth, 0.42 atmospheres inspired oxygen pressure) (94.3 ± 3.6%), it can be concluded that breathing a hypoxic air mixture does not induce a significant decrease of FMD potentially associated with vascular dysfunction, even in oral breathing conditions (Figure 2).

### 3.2. ROS Production and Lipid Peroxidation

**Figure 3:**
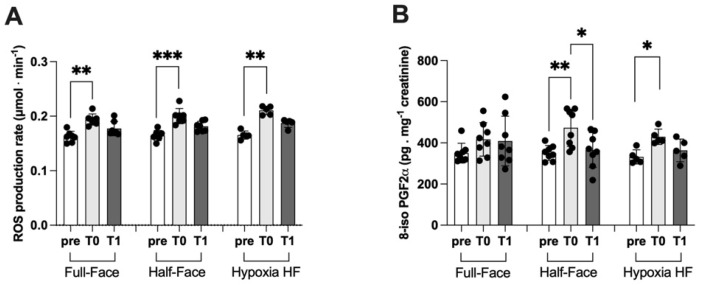
Panels show the histogram plot (mean ± SD) of (**A**) ROS production (EPR), and (**B**) 8-iso PGF2-α concentration (ELISA). * *p* < 0.05, ** *p* < 0.01, *** *p* < 0.001 significant difference.

In association to a shallow dive, independent of the mask worn and the air mixture, ROS production, assessed by means of the EPR detection of the Spin probe CMH, was significantly increased on return to the surface (T0). ROS production increased from 0.16 ± 0.01 to 0.19 ± 0.01 μmol⋅min^−1^ and from 0.16 ± 0.01 to 0.20 ± 0.01 μmol⋅min^−1^ when wearing a full-face mask and a half-face device, respectively. Breathing a hypoxic mixture utilizing a half mask, ROS production increased from 0.16 ± 0.01 to 0.21 ± 0.01 μmol⋅min^−1^ (Figure 3A). In all cases, a trend to a recovery to the predive value was observed at T1 corresponding to 3 h from surface, without reaching a significant level within the short window of experimental time.

Similarly (Figure 3B), the concentration of 8-iso PGF2-α, a marker of lipid peroxidation, was significantly increased after the dive session wearing a half-face mask. When breathing atmospheric air, values increased from 351.10 ± 36.35 to 473.40 ± 90.06 pg·mg^−1^ creatinine, returning to 364.90 ± 84.50 pg·mg^−1^ creatinine within the experimental window of time. A significant increase (331.90 ± 34.16 vs. 430.0 ± 37.35 pg·mg^−1^ creatinine) associated with a shallow dive was also observed when subjects were submitted to hypoxia while wearing a HF device. Additionally, in this case we observed a trend toward recovery after 3 h from surfacing (363.50 ± 54.80 pg·mg^−1^ creatinine), which was not significant within the observation window of time. On the other hand, no significant changes were observed when wearing a full-face mask. 

### 3.3. Nitric Oxide Synthase and Nitric Oxide Metabolites

**Figure 4:**
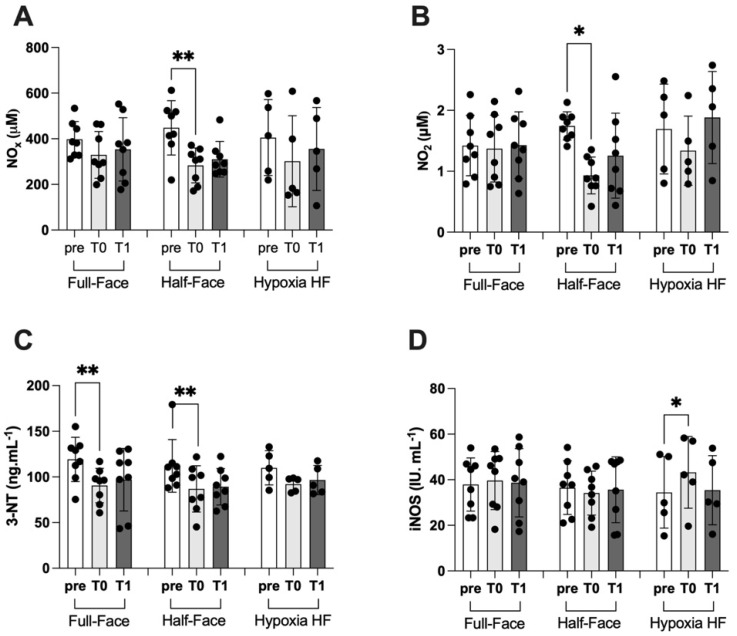
Panels show the histogram plot (mean ± SD) of (**A**) NO metabolites (NOx) (ELISA), (**B**) nitrite (NO_2_) (ELISA), (**C**) nitrotyrosine (3-NT) (ELISA) and (**D**) inducible nitric oxide synthase (iNOS) (ELISA). * *p* < 0.05, ** *p* < 0.01 significant difference.

As mentioned in the introduction, oral breathing can significantly reduce circulating nitric oxide (NO), possibly promoting in turn the basis for an endothelial dysfunction [8]. Considering the observed decrease of nitrotyrosine levels when wearing either the full-face or the half-face mask (Figure 4C), we sought to assess the presence of a parallel modulation of nitric oxide metabolites and of nitric oxide synthase activity.

We observed a significant modulation of both nitric oxide metabolites (NOx) and, more specifically NO_2_ levels, only in subjects wearing the half-face mask but not the full-face mask. Similarly, a shallow dive performed while breathing a hypoxic mixture was not associated with significant changes in NOx and NO_2_ levels. The time after surface was not associated with significant changes of both NOx and NO_2_, within the temporal window of time considered (Figure 4A,B).

Plasma levels of nitrotyrosine, a product of tyrosine nitration mediated by reactive nitrogen species such as peroxynitrites and nitrogen dioxide and therefore identified as a marker of cell damage and inflammation, significantly decreased at the end of a shallow dive both when wearing a full-face and a half-face mask. At 3 h from surface there was still no full recovery to baseline values except during hypoxic breathing, when no significant changes were observed (Figure 4C).

Interestingly, we observed no significant variations in the activity of iNOS, the inducible form of nitric oxide synthase, in association with a shallow dive performed by utilizing both the half- and the full-face mask. On the other hand, and somehow surprisingly, we observed a significant increase of iNOS activity at the surface, which is not attributable to a positive transcriptional regulation, being the diving time too short to justify a de novo synthesis. In all cases, no significant variations of iNOS activity were observed at 3 h from surfacing (Figure 4D).

## 4. Discussion

As a preliminary tenet to the discussion of our results, it is important to remark that we do not attribute a necessary negative value to alteration specific biochemical/physiological parameters, characterized by the involvement of a huge number of variables and homeostatic switches. These should be, at least initially, considered and interpreted as “response” and not as “biomarkers for an occurring damage”.

This is the case of the transient significant decrease of FMD observed after a SCUBA dive and, in particular, when performed while wearing a mask that allows breathing only through the mouth. Therefore, rather, we have utilized this parameter as a sensitive indication for a different physiological response to high oxygen breathing performed according to two different experimental modalities.

Sex differences in endothelial functions, and therefore on FMD response, have been reported and considered a confounding variable that amplify the within-group variability [37]. However, the number of subjects enrolled in the study was based on the variability observed in previous studies from our laboratory and others. We assumed a similar effect on FMD response after diving (roughly 5–7%), which is indeed comparable with a commonly accepted span observed in a specific sex group.

Newly published data indicate that FMD variations can be related to circulating microparticles [35], which were not considered in the study reported herein. FMD after hypoxic breathing showed a trend to increase, therefore suggesting that the influence of hypoxia-triggered microparticle formation is limited in our setting and, possibly, a greater influence of inspired NO.

Our results on FMD variation after a dive that was not prone to produce bubbles are comparable to previously published data from our group and others, although bubbles have been occasionally observed within different experimental settings [2,5,37]. Interestingly, previous studies have also demonstrated the occurrence of endothelial dysfunction after breath-hold diving [9,36,38]. This effect was prevented by nutritional supplementation of molecules putatively having either an antioxidant activity or affecting endogenous machinery involved in redox regulation [9,10,38]. These observations suggest that the effects of diving on FMD are possibly due to a decrease of NO generation or bioavailability [38,39]. It is plausible that an exceeding generation of reactive species could interact with NO, subtracting it to the vascular function and increasing the condition of oxidative stress induced by the increased partial pressure of oxygen at depth. This situation is present in SCUBA divers and breath-hold divers, but slightly different mechanisms are involved. Noteworthy, several studies have already addressed the effect of restricting breathing by mouth on respiratory and cardiovascular parameters, NO production and metabolism at surface, not associated with underwater diving or, generally, to either hyperoxia or hyperbaric oxygen exposure, suggesting that nasal breathing plays an important role in vascular performances [40,41].

After a shallow dive, we observed a significant increase of ROS generation in all conditions tested, eventually returning at baseline levels at 3 h from reaching the surface (T1). An increase of ROS generation is less expected after hypoxia, however; this observation is in agreement with several previous studies indicating that low oxygen concentration induces dysregulation of cellular oxygen metabolism and increased generation of ROS and 8-iso PGF2-α [42,43] in nonadapted subjects. However, these acute variations of oxidative status seem not to remain for long and return back to baseline within three hours (T1), after all exposures of diving-associated hyperoxia.

In agreement with the hypothesis described above, both hyperoxic and hypoxic exposure have been reported to induce an increase of oxidative stress [42]. ROS production significantly increased in all experimental conditions. The lesser increase observed in dives with a full-face mask (+15%) with respect to the half-face mask (+25%) and hypoxia wearing a half-face mask (+31%). This observation supports the hypothesis that nasal breathing, inducing NO production, could counteract ROS oxidative stress (Figure 3). Accordingly, we observed a significant increase of the marker of lipid peroxidation 8-iso PGF2-α when diving was performed using the classical half-face mask, both breathing hyperoxic or hypoxic mixture at depth but not when wearing a full-face device.

It has been previously reported [44], that high levels of oxygen breathing acutely alter NOS-dependent NO generation in healthy subjects. In our study, we observed a significant reduction in plasmatic levels of NO metabolites (NOx and NO_2_, respectively, in Figure 4A,B), which are widely accepted as markers of systemic NO generation. This reduction was evident and more pronounced in subjects wearing the half mask than in those utilizing the full-face device (−37 vs. −17% respectively). This difference confirms that nasal breathing hinders, at least in part, the impairment of NO availability, possibly due to the interaction with ROS, thus preventing endothelial dysfunction as measured by FMD [38].

A dive session performed at 10 m and lasting 20 min was not associated with any significant variation of iNOS activity when the subjects were wearing either a full-face mask or a half-face mask. Conversely, when the dive was conducted in slightly hypoxic conditions (16% oxygen at depth), wearing a classical half-face mask, we observed an increase of iNOS expression and activity, suggesting hypoxia and not hyperbaria is the factor inducing the upregulation of iNOS expression. This result is in agreement with previous studies showing that oxygen deprivation results in a significant increased expression of iNOS due to the activation of the nuclear transcription factor kappa B (NF-κB) [45,46]. Two points remain open to further investigation: first is in the extreme rapidity of iNOS induction in our experimental condition [47]. The second is that the increase of iNOS after hypoxia is not accompanied by an increase of NO metabolites. We hypothesized that the hyperoxic breathing during diving associated with an increase of ROS generation might have led to a decrease of NO availability and therefore to a decrease of circulating NOx [48].

It is well known that NO production at vascular level relies on two different mechanisms: an inducible synthase (iNOS) expressed upon the activation of a specific set of redox sensitive transcription factors, NF-κB possibly being the most important, and a constitutive one, the endothelial form eNOS, mainly regulated by Ca availability. We previously reported that variations of the composition and pressure of air breath are associated with a specific pattern of activation of oxygen-sensing transcription factors, including NF-κB, NRF2 and HIF [49]. In the present study, targeting the effect of nasal breathing on NO production as related to oxidative stress due to increased oxygen partial pressure, we focused our attention on the inducible form, but we also did not exclude that eNOS activity could be affected by a temporary alteration of intracellular Ca availability from the endoplasmic reticulum [50]. iNOS expression after a dive performed while breathing a hypoxic air mixture and wearing a mask that only allows mouth breathing can be due to the activation of NF-κB transcription factor, as confirmed by several publications [51]. Conversely, in the presence of basic hyperoxia, a more complex response, including a Ca release from endoplasmic reticulum, is likely to occur [52].

Accordingly, we observed a decrease of 3-NT in hyperoxia, irrespective if breathing through a half-face or a full-face mask, but not in hypoxia. Peroxidative nitration by nitrite and H_2_O_2_ forms 3-NT [53,54], which, in turn, needs superoxide ion to be produced. Lower 3-NT levels can be directly associated with the decreased nitrite level observed while wearing half-face mask and possibly lower ROS levels observed while wearing a full-face mask.

An increase of 3-NT levels is usually interpreted as an index of redox imbalance. In fact, 3-NT is a product of tyrosine nitration mediated by reactive nitrogen species. In our experimental conditions, we observed a decrease of NO metabolites (Figure 4A,B) in divers breathing from mouth only and no significant variations when using a full-face mask or in hypoxic conditions. Therefore, we can speculate that nitrotyrosine, which is formed in the presence of peroxynitrite, mainly generated following the reaction between NO and superoxide, are reduced accordingly. The increase of ROS generation assessed by means of EPR technology does not allow discrimination between different reactive oxygen species. As also mentioned above, we hypothesize that the predominant species are superoxide in hyperoxia and hydroxyl radical during hypoxia, due to metabolic production [54].

In our experimental setting, we acknowledge a ROS increase regardless of oxygen exposure. Nevertheless, we can hypothesize that the amount of ROS produced during the hypoxic period would mainly originate from metabolism, since exogenous oxygen is reduced. In this perspective, we can assume that a relatively higher superoxide ion generation occurs during hyperoxia and more hydroxyl radicals during hypoxia, due to metabolic production [54]. This will result in an increase of 3-NT during hyperoxic periods but not during hypoxia (Figure 4C). Moreover, 8-iso PGF2-α is increased during hypoxia and during hyperoxic oral breathing (HF-mask) marking increased lipid peroxidation, which depends more on the availability of hydroxyl radicals. During HF hyperoxic breathing, the increased isoprostane can be due to the higher generation of ROS concomitantly demonstrated by the increased lipid peroxidation associated with NOx and NO_2_ reduction.

As mentioned, hyperoxic breathing is likely to be associated with an increase in ROS and, therefore, the reduction in NO bioavailability is probably due to the formation of superoxide, which reacts with NO to form peroxynitrite, which in turn, nitrates proteins to form 3-NT. However, our previous studies indicate that peroxynitrite reaction associated with diving does not always follow the expected pattern [9,22]. Accordingly, other researchers found a similar pattern of NOx levels in blood samples obtained both underwater and after diving [55]. These observations highlight the evidence that other “actors” and mechanisms are involved in NO metabolism under modified oxygen concentration and under an altered respiratory pattern.

It should also be noted that any organism’s response to a stimulus (in this case diving and forced oral breathing) is not likely to be placed “exactly” at surfacing. Rather, a progressive adaption to a contingent environment occurs, involving a systemic response. Time points adopted in our study plan must be considered indicative “reference points”. It is therefore possible to hypothesize that there is no perfect synchronicity between ROS and NO generation: while the former is a “continuous”, starting from the exposure to high oxygen (independently of nasal or oral breathing), iNOS activation requires the transfer of signals through an oxygen sensor to sensitive transcription factor (NF-κB) and, lastly, protein synthesis. Finally, it should also be considered that other possible pathways allowing NO elimination exist besides the formation of peroxynitrate, which are impossible to be tested in an in vivo clinical study, possibly leading to a blunted and unexpectedly unchanged 3-NT production in the presence of hyperoxia [56].

## 5. Conclusions

Our study indicates that evident endothelial reactions occur while diving and that endothelial function can be “protected” using a full-face mask that allows nasal breathing. Moreover, a reduction of oxygen partial pressure attained by breathing a hypoxic (16% O_2_ at depth) countered FMD decrease even when only oral breathing was allowed. It is therefore very likely that oxidative stress is one, if not the unique, determinant of endothelial NO production (or more available) by nasal breathing and it is directly responsible for maintaining the function, probably by ROS scavenging activity. This phenomenon has already been described to explain hyperoxic vasoconstriction [57,58]. In fact, the vasodilatory effect of superoxide dismutase in hyperoxia was not seen in animals given prior doses of the NO synthase inhibitor [58]. These results provide evidence that one mechanism for hyperoxic vasoconstriction in the brain consists of inactivation of NO by superoxide anions, decreasing its basal vasorelaxing action. The direct physiological response is clearly present, although the biochemical markers are less evidently triggered, and their response is still partially blunted.

To our knowledge, this is the first study describing these aspects of diving physiology and, therefore, more investigations are needed to better understand this apparent difference in the response time. However, besides the obvious outcomes in terms of SCUBA diving safety, our data permit a better understanding of the effects of oxygen concentrations, either in normal conditions or as a strategy to induce selected responses in health and disease.

## Data Availability

Data are available from the authors by request.

## Abbreviations

EPO	Erythropoietin
EPR	Electron paramagnetic resonance
FMD	Flow mediated dilation
GSH	Intracellular reduced glutathione
HPLC	High-performance liquid chromatography
iNOS	Inducible nitric oxide synthase
8-iso PGF2-a	8-Isoprostane
MPPs	Matrix metallopeptidases
NF-κB	Nuclear factor kappa-light-chain-enhancer of activated B cells
NRF_2_	Nuclear factor (erythroid-derived 2)-like 2
NO	Nitric oxide
NO_2_	Nitrite
NOx	NO metabolites (NO2+NO3)
3-NT	Nitrotyrosine
PO_2_	Oxygen partial pressure
ROS	Reactive oxygen species
